# Impact of Work Environment and Occupational Stress on Safety Behavior of Individual Construction Workers

**DOI:** 10.3390/ijerph17228304

**Published:** 2020-11-10

**Authors:** Minhyuk Jung, Soram Lim, Seokho Chi

**Affiliations:** 1 Department of Architecture and Architectural Engineering, Seoul National University, 1 Gwanak-Ro, Gwanak-Ku, Seoul 08826, Korea; archidea914@snu.ac.kr; 2 Department of Construction Science, Texas A&M University, 3137 TAMU, College Station, TX 77843, USA; soramlim@gmail.com; 3 Department of Civil and Environment Engineering, Seoul National University, 1 Gwanak-Ro, Gwanak-Ku, Seoul 08826, Korea

**Keywords:** safety behavior, work environment, occupational stress, structural equation modeling, construction worker

## Abstract

This study was conducted to investigate how the work environment and psychological state influence construction workers’ perceptions and safety behaviors. Structural equation modeling was developed with five factors on the working environment (i.e., job demand, job control, job support, rewards, organizational justice, lack of reward), two factors on workers’ psychological condition (i.e., depression and trait anxiety), and four factors on safety perception (i.e., safety motivation, safety knowledge, and safety compliance and participation behaviors). Sample data were collected from 399 construction workers working at 29 construction sites in South Korea and analyzed the direct and indirect effects between those factors. The results showed that construction workers’ safety compliance and participation behavior are related to their safety knowledge and motivation, and depression and trait anxiety were found to lower safety motivation, knowledge, and, eventually, safety behavior. Job demands, lack of job control, lack of reward, and lack of organizational justice negatively impacted safety behavior. In contrast, job support did not show a significant relationship with safety behavior.

## 1. Introduction

The construction industry has traditionally been one of the sectors with the highest number of work-related injuries and fatalities. According to a report published by the Ministry of Employment and Labor in South Korea [1], 24,718 (30.6%) out of the 80,665 occupational accidents of the entire industry occurred only in the construction industry, and 506 (52.5%) out of 964 deaths occurred at construction sites in 2017. Compared to the other manufacturing industries that perform production activities based on automated factory systems, most of the construction industry’s production activities are being performed manually by human workers [2]. However, as workspaces in construction sites change as construction progresses, it is challenging to install permanent safety equipment and facilities that can protect construction workers from accidents, such as falling and electrocution. Moreover, working spaces and moving paths of construction workers often overlap with those of heavy equipment [3,4], which exposes workers to hazards, such as struck-by and caught-In/between accidents. Therefore, one of the best ways to prevent accidents at construction sites is to raise individual workers’ awareness of safety and to make them perform their works safely at all times [5].

Previous studies have made efforts to identify factors that influence safety accidents at construction sites and establish their relationships [4,6,7]. However, those studies have mainly focused on the impact of organization-level factors (i.e., safety climate and safety training programs) on construction organizations’ safety performance. These studies have provided useful insights into what management measures should be taken to reduce safety accidents. However, accident occurrences at construction sites can be better explained by differences in individual construction workers’ perceptions and behaviors about safety because construction sites continuously change dynamically. Therefore, it is difficult to control all the construction sites’ situations with organizational efforts at the organizational level. Consequently, it is crucial to identify factors affecting individual differences in safety behaviors and understand their relationships.

Furthermore, the behavior of individuals can be influenced by the emotions and knowledge regarding that behavior [8], their psychological condition [8,9,10], and the safety of the environment surrounding them [11,12]. These factors are often interrelated; therefore, they can affect an individual’s safety behavior in various ways. Consequently, it is essential to analyze the relationship between those factors surrounding the individual’s safety behavior simultaneously to understanding how they affect safety behavior. Nevertheless, many studies have investigated a limited number of factors, and the knowledge gap resulting from this can limit the range of management that construction managers can choose to improve workers’ safety behavior.

Therefore, this study examines the relationship between working environment-related factors and workers’ psychological factors with construction workers’ perceptions and behaviors on safety. First, this study reviewed previous studies to derive environmental and psychological factors affecting construction workers’ safety behavior, and based on the literature review, research hypotheses and models were established. Second, sample data were collected to verify the research hypothesis through a self-questionnaire survey and applied to a statistical model based on a structural equation model to confirm direct and indirect effects between the derived factors. Finally, based on the analysis results, the validity of the hypothesis was determined, and the academic and practical implications of the research result were discussed. Through this process, this study will provide construction site managers with the information necessary to raise safety awareness and behavior of individual workers by clarifying the relationship between environmental, psychological related to safety awareness and behavior.

## 2. Literature Reviews

### 2.1. Safety Perception and Behavior of Individual Workers

Griffin and Neal [13] developed a safety performance model concerning performance theory [8,10] to determine how perceptions of safety-related organizational factors (e.g., safety climate and safety training program) differ from individual perception on safety motivation, knowledge, and behaviors. Campbell’s performance theory [8] is a model that describes actual behaviors that individual workers perform in their workplaces using proximal and distal factors that affect these behaviors. This model consists of three parts: components of performance, determinants of performance, and performance antecedents.

The components of performance, first, refer to work-related behaviors of individuals, which are divided into two concepts on performance: (a) task performance, which relates to a worker’s formal and core responsibilities; and (b) contextual performance, which does not correspond to a worker’s core activity but can contribute to the development of a social and organizational environment that can facilitate the core activity of the organization [14,15]. Griffin and Neal [13] explained the task performance and contextual performance in a safety context using safety compliance and safety participation concepts. Safety compliance is defined as the degree to which workers follow safety manuals and work safely, and safety participation is related to a worker’s voluntary efforts to develop a safe environment and reduce potential hazards in workplaces.

Next, the determinants of performance include direct causes of individual differences in work performance, and Campbell et al. [8] suggested three determinants to affect work performance: knowledge, skill, and motivation. Based on these factors’ definitions, Griffin and Neal [13] suggested safety motivation and safety knowledge as determinants of the worker’s safety behaviors (i.e., safety compliance and safety participation). The former is a term that indicates whether workers are concerned with safety, and the latter explains whether workers know the correct ways to perform their work safely [16].

The antecedents of performance are external factors that can affect work performance, mostly mediated through knowledge, skills, and motivation. The antecedents of performance are divided into organizational and individual-level factors [9,17]. The organizational-level factors include organizational climate and culture and working environment and condition, while the individual-level factors are related to workers’ ability, experience, temperament, and psychological condition. Griffin and Neal [13] studied how safety climate affects safety behaviors based on his model; thus, they found a significant distinction between the organization-level perception of safety and individual workers’ perception of safety motivation, knowledge, and behaviors. Moreover, Probst and Brubaker [16] examined that job security and job satisfaction, which are psychosocial factors on the working environment, significantly impacted workers’ safety motivation, knowledge, and behavior in the food-processing industry.

As such, many studies have been conducted on the safety behavior of workers in various industries. However, studies on the safety of construction sites have mainly focused on the relationship between the organizational-level factors (e.g., organizational culture and climate) and the organizational-level safety performance (e.g., near-miss, injury). Few studies have attempted to determine which factors affect safety behavior. Li et al. [18] studied the effect of job demand and job resources on the number of near-misses and injuries. They used the concept, safety compliance as a mediation variable. Leung et al. [19] analyzed how the work environment and safety equipment are related to injury incidents via construction workers’ stress and safety behavior. However, they did not explain how work environment factors influence personal safety behavior.

Therefore, in the current study, as proposed by Neal and Griffin, the safety behavior of construction workers is described in terms of two concepts of safety compliance behavior and safety participation behavior. Besides, it is assumed that the motivations and knowledge of individual construction workers’ safety each act as the determinants of their safety behaviors. Therefore, this study establishes the following hypothesis:

 **Hypothesis 1 (H1).** *Safety motivation and knowledge of construction workers directly affect their compliance behavior and participation behavior on safety.*

 **Hypothesis 1 (H1a).** *Safety motivation increases safety compliance behavior.*

 **Hypothesis 1 (H1b).** *Safety motivation increases safety participation behavior.*

 **Hypothesis 1 (H1c).** *Safety knowledge increases safety compliance behavior.*

 **Hypothesis 1 (H1d).** *Safety knowledge increases safety participation behavior.*

In addition, this study analyzes how the work environment of construction sites, as antecedents at the organizational level, and the psychological condition of workers, as antecedents at the individual level, affects safety compliance and participation behaviors in the following sections.

### 2.2. Psychosocial Factors on Work Environment

Many previous studies have been carried out on work environment-related psychosocial factors and workers’ safety behavior. First, one of the most influential models that directly explains the impact of the working environment on a worker’s psychological condition and behavior is the demand-control (DC) model [20]. The DC model’s premise is that work-related benefits and satisfaction depend upon a reciprocal relationship between job demand and job control. The former signifies the requirements set at work, and the latter means controlling and organizing their work. For example, workers with low job control and high job demands have low satisfaction with their jobs [21,22] and are more likely to have stress-related diseases such as high blood pressure [23].

About ten years after the DC model was developed, Johnson and Hall [24] extended the model by adding the concept of job support, which means social isolation and interpersonal conflict in workplaces. Many empirical studies have been conducted based on this model and shown that the demand-control-support (DCS) model factors are significantly related to workers’ psychological and physical health [25] and work-related motivation, behaviors, and various aspects of performance. Many previous studies have been conducted on the relationship between the DCS model and safety performance [18,26,27]. Bronkhorst [28] has found that high job demand negatively affects healthcare staff’s safety compliance and safety participation.

The effort-reward imbalance (ERI) model [29,30] is another representative model in the occupational health psychology domain studying the impact of the working environment and condition on workers’ health. According to this model, the psychosocial working environment can be characterized by a combination of job demands and rewards [29,31]. For example, workers who perceived low rewards compared to their commitment showed lower physical and mental health [30] and differed in job performance-related behaviors, such as absenteeism and tardiness [32].

The concept of rewards in workplaces are generally classified into financial reward (e.g., salary), psychological reward (e.g., respect and support), and job opportunity (e.g., job security and advancement) [31]. Probst and Brubaker [16] found that low job security could lead to impairment of safety-related behaviors by mediating job satisfaction. Smith et al. [33] also mentioned that employment security could have a significant impact on safety climate and individual employers’ safety behavior.

Another work environment-related psychosocial factor that can influence safety behavior is organizational justice. Leventhal [34] argued that employers’ perceptions of organizational justice had a significant impact on their work attitudes and performance, and Zohar argues that organizational fairness is closely related to the organization’s atmosphere. Skarlicki and Folger [35] classified organizational justice into three concepts: distributive, procedural, and interactional justice. According to his study, distributive justice implies worker’s perception of fairness in allocating organizational resources, procedural justice in work-related decision-making procedures, and interactive justice in an interpersonal relationship with their supervisors. Gyekye and Haybatollahi [36] provide evidence that a worker’s perception of organizational fairness has a significant relationship with workplace safety perceptions. Moreover, Marín et al. [37] noted that abusive supervision could negatively affect occupational diseases and injuries. Although studies on the relationship between organizational fairness and safety behavior have not been carried out as much as the aforementioned work environment-related factors, it can be inferred from its impact on work-related behavior and injury that organizational justice may be related to workers’ safety behavior.

This study examines how the five work environment factors mentioned above (i.e., job demand, job control, job resource, job rewards, organizational justice) affect construction workers’ safety behavior. To this end, as Neal and Griffin have argued, work environment-related factors were set as antecedents of safety behaviors. The hypotheses established are as follows:

 **Hypothesis 2 (H2).** *Work environment factors at construction sites affect construction workers’ safety compliance and participation behaviors through their safety motivation and knowledge.*

 **Hypothesis 2a (H2a).** *Increased job demand reduces safety compliance and participation behaviors via safety motivation and knowledge.*

 **Hypothesis 2b (H2b).** *Decreased job control reduces safety compliance and participation behaviors via safety motivation and knowledge.*

 **Hypothesis 2c (H2c).** *Decreased job support reduces safety compliance and participation behaviors via safety motivation and knowledge.*

 **Hypothesis 2d (H2d).** *Decreased rewards reduce safety compliance and participation behaviors via safety motivation and knowledge.*

 **Hypothesis 2e (H2e).** *Decreased organizational justice reduces safety compliance and participation behaviors via safety motivation and knowledge.*

On the other hand, in order not to rule out the possibility of a direct effect of work environment factors on the safety behaviors, the following additional hypotheses are set:

 **Hypothesis 3 (H3).** *Work environment factors at construction sites directly affect construction workers’ safety compliance and participation behaviors.*

 **Hypothesis 3a (H3a).** *Increased job demand reduces safety compliance and participation behaviors directly.*

 **Hypothesis 3b (H3b).** *Decreased job control reduces safety compliance and participation behaviors directly.*

 **Hypothesis 3c (H3c).** *Decreased job support reduces safety compliance and participation behaviors directly.*

 **Hypothesis 3d (H3d).** *Decreased rewards reduce safety compliance and participation behaviors directly.*

 **Hypothesis 3e (H3e).** *Decreased job organizational justice reduces safety compliance and participation behaviors directly.*

### 2.3. Psychological Factors of Construction Workers

Many previous studies [38,39,40] have analyzed various psychological factors to derive individual-level factors that can influence construction workers’ motivation, knowledge, and behavior. Psychological factors that have been mainly dealt with in these studies include depression, state anxiety, nervousness, extroversion, and communication materials. This study focuses on depression and trait anxiety, which have been mentioned as having a significant relationship with the work environment [30,32] while affecting a worker’s safety awareness and behavior [39,40].

Depression, referred to as a major depressive disorder, is a long-term mood disorder characterized by a feeling of sadness and loss of interest [41]. Many studies [42,43] have consistently found that depression is related to deficits in verbal and visual memory and learning. Besides, depression was found to harm motivation by reducing human’s ability to perform necessary activities voluntarily or in response to external factors [44,45]. Depression, which has a significant influence on people’s perceptions and behaviors, has also been shown to affect work performance [43]; in particular, it has been shown to affect workplace safety negatively. For example, Beseler and Stallones [46] found that farm operators and their spouses, who have high levels of depression but have low levels of safety knowledge, showed a higher incidence of injury. In addition, depression is a factor that affects safety-related factors and one of the significant mental health factors that are affected by the work environment. Notably, depression has been reported to have a high possibility of being caused by the imbalance of job demand and job control [20,25].

Anxiety, similar to depression, is another major factor that is dealt with in occupational health psychology and is characterized by feelings of anxiety and fear [47]. Spielberger [48] explained anxiety by dividing it into state and trait anxiety. The state anxiety is a state of temporary and situational emotion that responds to threats and can be felt by anyone in everyday life. On the other hand, trait anxiety is a personality trait associated with feeling worrying and anxious quickly. People with high levels of trait anxiety feel anxious by recognizing a broad range of environmental events as potential threats. Meijer [49] found that people with higher trait anxiety respond to self-esteem threats with a higher level of state anxiety, but no significant difference was shown with physical hazards and threats [48].

The impact of job insecurity on work performance has been addressed in many previous studies. However, they have shown contradictory results. A study on sales assistants [50] showed that high anxiety workers have no difference in productivity but much higher sales performance by exerting more work effort. On the other hand, Kanfer and Heggestad’s study [51] found that people with higher anxiety had lower self-regulation that controls the emotions needed to keep their attention to work, resulting in lower work performance. Reio and Callahan [52] found that high trait anxiety impaired an individual’s curiosity, socialization-related learning, and work performance.

Furthermore, trait anxiety has been shown to have a negative effect on safety behavior and the incidence of injuries. Dunbar [38] found that workers with a high level of anxiety increased behaviors that did not wear personal safety equipment and did not comply with safety-related rules. One explanation of these results by Probst and Brubaker [16] is that job insecurity had a negative effect on attitudes toward work in the form of anxiety, which in turn resulted in lower safety performance. In addition, Murray et al. [53] revealed that fishers with high anxiety levels showed more injuries and carelessness. Like depression, anxiety is known as one of the traditional psychological stressors significantly influenced by the work environment [54].

The previous studies mentioned above have found that employees’ depression and anxiety symptoms can significantly affect safety behaviors. Therefore, this study hypothesizes that depression and trait anxiety will affect safety behaviors as antecedents mediated by safety motivation and knowledge:

 **Hypothesis 4 (H4).** *Construction workers’ psychological state affects their safety compliance and participation behaviors through safety motivation and knowledge.*

 **Hypothesis 4a (H4a).** *Increased depression reduces safety compliance and participation behaviors via safety motivation and knowledge.*

 **Hypothesis 4b (H4b).** *Increased trait anxiety reduces safety compliance and participation behaviors via safety motivation and knowledge.*

On the other hand, as with the work environment factors, little evidence has been presented in previous studies that depression and anxiety will not affect safety behaviors through pathways other than safety motivation and knowledge. Therefore, this study sets the following hypothesis:

 **Hypothesis 5 (H5).** *Construction workers’ psychological state directly affects construction workers’ safety compliance and participation behaviors.*

 **Hypothesis 5a (H5a).** *Increased depression reduces safety compliance and participation behaviors directly.*

 **Hypothesis 5b (H5b).** *Increased trait anxiety reduces safety compliance and participation behaviors directly.*

Depression and anxiety affect work and safety performance and are also known to be factors induced by the work environment-related factors. Therefore, this study’s model includes the effects of depression and trait anxiety on the construction environment’s safety behavior. The relevant hypotheses are as follows:

 **Hypothesis 6 (H6).** *Construction workers’ psychological state mediates work environment factors on safety behaviors.*

 **Hypothesis 6a (H6a).** *Depression mediates the effect of work environment factors on safety behaviors.*

 **Hypothesis 6b (H6b).** *Trait anxiety mediates the effect of work environment factors on safety behaviors.*

### 2.4. Research Model

**Figure 1:**
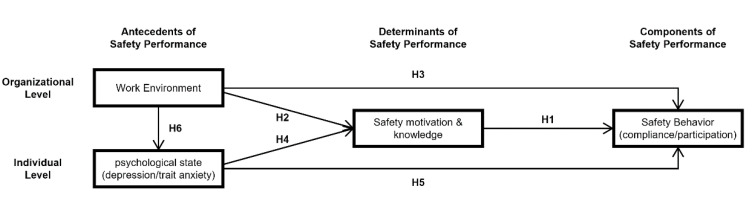
Conceptual research model.

This study analyzes the relationship among construction workers’ perception of the work environment at construction sites, their psychological status, safety motivation and knowledge, and safety compliance and participatory behavior. A research model based on the hypotheses mentioned above is shown in Figure 1. The research model analyzes complicated relationships among the work environment’s five factors, two factors of a psychological condition: safety motivation and knowledge, and two factors of safety behavior. Based on the method, the non-significant model is sequentially removed to obtain a meaningful model. Therefore, this paper derives the final model by sequentially removing the insignificant relationship using the experimental method.

## 3. Research Methods

### 3.1. Measurement

#### 3.1.1. Work Environment

A questionnaire was developed to measure the six work environment-related factors (i.e., job demand, job control, job support, lack of reward, and organizational justice) that could affect the safety behavior of construction workers, referring to the Job Content Questionnaire (JCQ) [55], effort-reward imbalance (ERI) [29], Occupational Stress Index (OSI) [56], and Job Stress Questionnaire (JSQ) of the National Institute for Occupational Safety and Health (NIOSH) [57]. The questionnaire items are described in Table 1. Each item is assessed on a four-point Likert-type scale ranging from 0–3, and the scores of questionnaire items of the four factors (job control, job support, lack of reward, and organizational justice) were reversely scored to match the direction of the relationship so that an increase in working environment factors would increase the negative impact on safety behavior. Reliability analysis and item-total correlation analysis were performed for the results obtained. Items with an item-total correlation of 0.4 or less and Cronbach’s alpha of 0.5 or less were removed [58]. As a result, two items were eliminated (marked in Table 1), and the subsequent analyses were conducted using these 20 items.

#### 3.1.2. Psychological Condition

The depression of construction workers was measured using the Center for Epidemiologic Studies-Depression (CES-D), which Radloff developed [59] to easily measure the symptoms of depression experienced by the general population. Many studies have shown that this tool has high reliability and is useful as a measurement tool for screening clinical patients with depression.

The CES-D questionnaire was originally developed with 20-item questions, each rated on a four-point scale with responses ranging from 0–3. However, too many numbers of questionnaire items increase the number of measurement variables, and it can increase the complexity of the statistical model and make it difficult to obtain correct statistical results. Therefore, this study used a four-item short-form, which was developed by Melchior et al. [60]. Melchior et al. [60] compared the results of 4 items and 20 items CES-D to prove the significant application of the short-form CES-D statistically. On the other hand, the construction workers’ anxiety state was measured using the State-Trait Anxiety Inventory (STAI) [47]. STAI is one of the most widely used tools for measuring anxiety symptoms and is comprised of STAI-X1 measuring state anxiety and STAI-X2 measuring trait anxiety. This study used STAI-X2, which consists of 20 items measured by the five-point scale from 0–5, and the sum of the item scores represents the degree of anxiety [61]. In this study, the six-item short-form of STAI, developed by Marteau and Bekker [62], was used. The questionnaire used to measure depression and trait anxiety of construction workers in this study is shown in Table 2. Reliability and item-total correlation analyses were carried out on the sampled data, and no item was removed.

#### 3.1.3. Safety Motivation, Knowledge and Behavior

Safety motivation, knowledge, and compliance and participant behaviors of construction workers were measured using the items suggested by Neal et al. [63], Neal and Griffin [64], and Vinodkumar and Bhasi [58]. Each factor consists of four questions, as described in Table 3. All questions were measured using a five-point scale ranging from 1–5. As with the other measuring instruments mentioned above, the reliability and correlation analysis was performed. However, all of the 16 items were adopted as the items that belong to each factor were found to be significantly related to each other.

### 3.2. Sample Data

Surveys were conducted to measure factors on the working environment, psychological condition, and construction workers’ safety awareness and behavior. A total of 430 questionnaires were distributed to 29 construction sites in South Korea, including housing, buildings, skyscrapers, roads, bridges, and tunnel construction sites. After removing questionnaires in which all the question items were not answered correctly, 399 valid responses were obtained, and thus the response rate of the questionnaire was 90.7%.

There is no consensus for the minimum number of sample data required to obtain valid statistical models’ results. However, many scholars have discussed this, and for a structural equation model (SEM), it is recommended that the sample size is larger than five times the free parameter. Considering the research model’s factor and path numbers, the saturated model, which assumed all paths, was found to have a maximum of 42 free parameters. Therefore, the minimum number of samples required for this research is 210, so this study’s sample number meets the criteria.

Demographic characteristics of the sampled data are shown in Table 4; 98% of all the respondents were male, and respondents over the age of 40 accounted for over 80% of the total. 63.4% of the total were daily laborers, and the rest were permanent workers or project-based contract workers. Around 80% of the population had more than five years of work experience, and about 60% had more than ten years of experience. Lastly, 7% of respondents had experienced a near miss or accidents while working in construction sites.

Table 5 shows the number of questions, measures, samples, maximum and minimum values, mean, and standard deviation for each variable.

### 3.3. Data Analysis

This study analyzes the measured data based on a structural equation modeling (SEM) technique. The SEM has advantages in that the regression coefficient can be estimated in consideration of the measurement error included in the measured items. Simultaneous estimation is possible in an interrelated-dependent relationship. It is possible to estimate the direct and indirect effects of various variables [65,66]. Therefore, this method is appropriate for analyzing the research model and the measured data of this study.

The structural equation model consists of a measurement model and a structural model [67]. The former establishes the reliability and validity of the observed variables based on hypothetical constructs, and the latter explores the relationship between the constructs.

The fitness of the two structural equation models and observed data is generally tested using a plurality of indices that evaluate a model based on various perspectives [68]. The fit indices of the SEM can be divided into three groups as follows. First, absolute fit indices describe how well sample data fit a research model. Well-used fit indices of this group include relative chi-square (χ^2^/df, df: the degree of freedom), root mean square error of approximation (RMSEA), and goodness-of-fit statistic (GFI). Second, indices belonging to the incremental fit indices represent fitness by comparing the chi-square value of the null model (i.e., baseline model), a model that all variables are unrelated. The related indices are the normed-fit index (NFI) and the comparative fit index (CFI). Finally, parsimony fit indices are relative indices developed to overcome the problem of lowering the fit index as the model becomes more complex and saturated. The parsimony goodness-of-fit index (PGFI) is one of these indices. However, these indices are recommended to be used in conjunction with other absolute and incremental indices, as they do not have recommended threshold levels. The acceptable threshold levels of the indices, as mentioned above, are shown in Table 6 [69,70].

For the measurement model, the construct validity is additionally tested, which verifies that the measurement tool properly measured the abstract concept. Construct validity can be tested through two types of validity: convergent validity and discriminant validity. The convergent validity assesses whether the measurement variables related theoretically and supposed to describe the same construct have a significant relationship. The convergent validity can be determined using (a) factor loading (acceptable level > 0.4) [67], (b) average variance extracted (AVE, acceptable level > 0.5) [66], and (c) construct reliability (CR, acceptable level > 0.7) [66]. By contrast, the discriminant validity determines whether no relationship exists between the supposed measures to be unrelated theoretically or to describe other contracts. If the AVE values are more significant than the latent variables’ squared correlation coefficient, the measurement model is considered to satisfy the discriminant validity.

**Figure 2:**
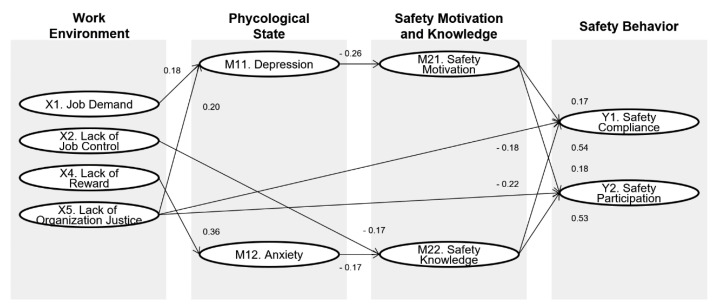
The final model.

The model for this study is organized hierarchically. A total of four high-level factors (i.s., work-environment, mental health, safety motivation and knowledge, and safety behavior) have been derived, and their theoretical relationships (Figure 2) were established through the literature review. Moreover, a total of 11 low-level factors known to be related to the high-level factors were derived. This study explores whether these low-level factors have a meaningful relationship through SEM analysis based on the collected data. Therefore, if necessary, the structural model is modified based on the fitness of the data.

## 4. Analysis Results

### 4.1. Measurement Model

First, the 16 observed variables of four factors related to safety motivation, safety knowledge, safety compliance, and safety participation were integrated into one measurement model, and a confirmatory factor analysis was conducted using AMOS 26 to confirm the fit and validity of the sample data with the measurement model. The fit indices of the model showed the acceptable levels as follows: χ^2^ = 1432.0 (*p* < 0.001); df = 835; χ^2^/df = 1.715; RMSEA = 0.042 (90% confidence interval (CI): 0.039–0.046); GFI = 0.856; NFI = 0.848; and CFI = 0.930.

Next, construct validity was tested. As shown in Table 7, the factor loading of all measures ranges from 0.506–0.893. In addition, the AVE values of the constructs range from 0.933–0.977, and the CR value from 0.979–0.994, which exceeds the acceptable levels. Therefore, it can be considered that convergent validity is established. Table 8 shows the correlation matrix of the latent variables. The squared correlation coefficient between latent variables shows the range of 0.316–0.704. Hence, the AVE value of all latent variables is more extensive than those values. Accordingly, the discriminant validity of the measurement model is established.

### 4.2. Structural Model

Based on the hypothesis model shown in Figure 1, a structural model was developed, and its fitness for use with the data was tested. The structural models showed goodness-of-fit indices: χ^2^ = 1409.4 (*p* < 0.001); df = 811; χ^2^/df = 1.738; RMSEA = 0.043 (90% CI: 0.039–0.047); GFI = 0.859; NFI = 0.850; CFI = 0.930; and PGFI = 0.736. However, since these figures are lower than the acceptable criteria, the hypothetical model suggested was found to be insufficient to analyze the relationships between the research variables. Thus, the hypothesis model was modified. In order to modify the hypothesis model, insignificant relationships among the variables were eliminated one at a time from the most insignificant one at a significant level. The modified model’s fit indices and the significance of the relationship between the variables were repeatedly confirmed when one relationship is removed. Then insignificant correlations between the exogenous variables were eliminated based on the covariance indicators. The final modified model is shown in Figure 2.

As shown in Figure 2, the lack of job supply (X3) was found to have no significant relationship with safety behavior variables. Hence, they were excluded from the final model. However, this does not mean that the concepts of the variables eliminated are irrelevant to the concepts of the other endogenous variables and are not valid within the sample and model presented in this study, but that they are not valid within the sample and model analyzed in this study. The fit indices of the final model is χ^2^ = 1432.0 (*p* < 0.001); df = 835; χ^2^/df = 1.715; RMSEA = 0.042 (90% CI: 0.039–0.046); GFI = 0.856; NFI = 0.848; CFI = 0.930; and PGFI = 0.732, which show improved fitness at all indices than the hypothetical model. Moreover, multiple correlation squares (SMC) of the final model were analyzed, which indicates the extent to which each endogenous variable of the model was accounted for by other variables. The results showed the depression of 0.058, the anxiety of 0.130, the safety motivation of 0.119, the safety knowledge of 0.141, the safety compliance of 0.493, and the safety participation of 0.517.

Figure 2 shows the significant relationships and path coefficients between the variables of the final structure model. First, the safety motivation was shown to have a positive relationship with the safety compliance (β = 0.169, *p* = 0.007) and the safety participation (β = 0.184, *p* = 0.004), and the safety knowledge with the safety compliance (β = 0.537, *p* = 0.000) and the safety participation (β = 0.533, *p* = 0.000). Therefore, since these all variables have significant relationships with each other, Hypothesis 1 is also fully satisfied.

The relationship between work environment and safety behavior was analyzed, and it was possible to see various relationships depending on the type of work environment. The lack of job control (X2) is the only variable that affects safety behavior being mediated by safety knowledge (β = −0.175, *p* = 0.000), as described in the model of Neal and Griffin [9]. Therefore, Hypothesis 2, which implies an indirect effect of the working environment on safety behavior, was partially satisfied; namely, only Hypothesis 2b was accepted. On the other hand, Hypothesis 3, which means a direct impact of the work environment on safety behavior, is only partially satisfied. The lack of organizational justice (H3e) was not mediated by the safety motivation and knowledge of construction workers but directly affected construction compliance (β = −0.181, *p* = 0.000) and participation (β = −0.218, *p* = 0.000) behavior. However, the other work environment-related variables were found not to affect safety behavior (H3a–d) directly.

The impact of construction workers’ psychological condition on safety behavior was analyzed. The depression was mediated by safety motivation (β = −0.263, *p* = 0.000) and trait anxiety mediated by safety knowledge (β = −0.168, *p* = 0.000) when they influenced safety compliance and participatory behavior. Accordingly, Hypothesis 4, which sets the role as antecedents of safety behavior, was found to be satisfied. On the other hand, Hypothesis 5, which means that depression and anxiety can directly affect safety behavior, was fully rejected. Meanwhile, Hypothesis 6, in which the psychological condition mediates the working environment’s impact on safety behavior, was partially adopted. The job demand (β = 0.180, *p* = 0.003) and the lack of organizational justice (β = 0.204, *p* = 0.003) indirectly affected safety behavior through depression (H6a), and the lack of reward (β = 0.364, *p* = 0.000) was mediated by anxiety (H6b).

The direct, indirect, and total effects between the two variables were analyzed based on the final model. Table 9 describes how the work environment variables and the psychological condition affect safety compliance and participation behaviors. The results show that the work environment variables and the psychological condition significantly impact safety compliance (Y1) and safety participation (Y2) in terms of the total effects. More specifically, the job demand (X1), lack of job control (X2), and lack of reward (X4) have indirect effects on safety behavior, and organizational justice (X5) has both direct and indirect effects on it. In addition, the psychological factors, the depression (M11) and the trait anxiety (M12), showed to have an indirect effect on safety behavior.

## 5. Discussion

### 5.1. Safety Motivation and Knowledge of Safety Behaviors

This study’s final model was found to fit relatively well with the collected data and showed statistically significant relationships between the variables among the four group factors.

Therefore, it is possible to explain the relationship between the working environment, psychological condition, safety motivation and knowledge, and safety compliance and participant behaviors.

First, safety motivation and safety knowledge appear to positively affect safety compliance and safety participation, respectively, as the previous studies’ models based on Neal and Griffin’s model [9] have shown. However, construction workers’ safety knowledge has a larger impact on both safety compliance and participation, while the motivation is relatively weaker.

In other words, the construction workers’ failure to perform their work safely or the correct safety procedures (i.e., safety compliance) is more influenced by their not knowing how to perform works safely than by not thinking that safety is essential in their workplace. In addition, construction workers’ less voluntary efforts to improve the work environment’s safety are more attributed to a lack of safety knowledge rather than a lack of awareness of its importance. This result shows the difference from other industries [13,17], where safety knowledge and safety motivation affect safety behavior at a similar level. One possible explanation for the reason why safety knowledge is more important than safety motivation to increase the safety behavior of construction workers is that their work environment, namely construction sites, changes significantly and continually compared to the production sites of other industries such as the service and manufacturing industries [71]. For example, unlike the manufacturing industry in which workers repeatedly work in the same spaces, construction workers work for the same type of construction work temporarily in one space and continue to move to another [72]. This may obscure workspaces’ boundary between different construction works and overlap their workspaces of workers and movement paths of heavy equipment [73,74]. Therefore, construction workers have to regain knowledge of their threats regularly.

Accordingly, even if construction workers are aware of the importance of safety, if they do not have sufficient knowledge of changing environments and other construction works possibly threatening their safety, construction workers can have the perception that they are not safely performing their work or that it is difficult for them to voluntarily behave to improve workplace safety due to the large variability and uncertainty on the construction site. Therefore, it can be considered essential to provide sufficient information about safety to construction workers to encourage their safety behavior.

### 5.2. Psychological Factors in Safety Behaviors

In the relationship of construction workers’ psychological condition with their safety behaviors, their depressed symptoms affected the safety compliance and safety participation via safety motivation. On the other hand, anxiety symptoms have an impact on them through safety knowledge. Accordingly, in terms of the total effect on the safety behavior, the effect of depression is relatively small compared to that of trait anxiety as it is mediated by the safety motivation, which has a smaller effect on safety behavior. Trait anxiety, on the other hand, has a relatively large total effect.

Many previous studies on human depression have reported that people with high depressive symptoms show lower ability in cognition, learning, memory, and motivation [42,44] than those with low depressive tendencies. However, the results in this study showed that depression has a significant relationship with only safety motivation but not safety knowledge. On the other hand, people with high anxiety are generally known to easily suffer cognitive fatigue when anxiety is concerned with excessive workload. In this case, excessive concentration on work can reduce attention and curiosity in other areas [52]. Similarly, the analysis results showed the construction workers with a high level of anxiety tended to have a negative effect on the acquisition level of safety knowledge, and consequently, on safety behavior.

Taken together, the two factors that indicate the psychological condition of construction workers, although different in their effects, were found to have a significant relationship with safety behavior. In particular, anxiety due to work could have a significant impact on construction workers’ safety behavior.

### 5.3. Working Environment in Safety Behaviors

Finally, the relationship between the factors of the construction site’s working environment and construction workers’ safety behavior was examined. The job demand influenced safety behavior by mediating psychological factors related to depression and motivation for safety. In other words, construction work with high workloads was found to have an effect on safety behavior by increasing depressive tendencies and decreasing safety motivation. This result is consistent with the previous studies [28,54] showing that excessive pressure on work increases depressive tendencies and reduces workers’ concentration on other factors not related to work performance. On the other hand, the high workload of construction workers did not significantly affect trait anxiety and safety knowledge. Accordingly, in construction sites with heavy workloads and tight schedules, it is crucial for managers to check for symptoms of depression and lowered safety motivation of the construction workers. This result is contrary to the findings of other industries’ previous studies [74,75] that production pressure can cause the acquisition of safety knowledge to be ignored. One possibility to explain these results is that the construction site is exposed to a certain level of unknown risk, regardless of the amount of work, because environmental changes and uncertainties of construction sites are large, and hazards and threats change dynamically.

The findings showed that the lack of job control was mediated by safety knowledge and indirectly affected safety behavior. In general, high job control means a higher level of job position, leading to a broader definition of the worker’s role, thereby increasing compliant and participatory safety behavior. In the results of this study, job control has indirect effects through safety knowledge. The organization of construction sites is hierarchically composed of contractors, subcontractors, and daily workers. Thus, the range of information and knowledge on construction sites that construction workers can learn is closely related to their job position. Therefore, as mentioned above, there can be a big difference in obtaining information about the interference between workspaces and the hazard location, which dramatically affects construction workers’ efforts to safely perform their work.

The lack of reward was then shown to influence safety compliance and participation behavior by mediating construction workers’ anxiety and safety knowledge. According to the ERI studies [30,31], lack of reward is one of the leading causes for reducing job satisfaction and increasing turnover rate. In other words, workers who lack compensation may ignore the factors necessary to continue working on a long-term perspective. However, when looking at the contract types of survey respondents, 63.4% of all workers are composed of low-paid daily workers. Therefore, the construction manager should make an effort to find a plan that allows workers to work in the long term or other ways to increase their job satisfaction.

Finally, the lack of organizational justice was found to affect safety behavior through two different paths. First, negative perceptions of organizational justice have a negative impact on safety behavior by increasing depressive tendencies and reducing safety motivation. In addition, organizational justice was shown to directly affect safety behavior, which can be attributed to reducing the propensity to comply with organizational rules and acting actively to improve rules when they feel unfair in the organization’s decision-making procedure, resource allocation, and information distribution. In terms of the total effect, organizational justice has the most significant impact on safety behavior than the other work environment factors and affects it directly. Therefore, efforts to improve organizational justice can be significant regarding workers’ safety behavior.

Overall, an individual worker’s safety behavior is related to a variety of internal and external factors. However, how these factors affect safety behavior are various because these factors have a complicated relationship with each other. The results on how these factors are related to each other can improve our understanding of workers’ safety behavior and provide useful knowledge necessary for construction sites’ managers to improve workers’ safety.

However, this study had the following limitations. First, this study tried to clarify the various factors that influence construction workers’ safety behavior and their relationships. However, the causality among the factors could not be confirmed, which means that hidden factors can explicitly explain these factors’ relationships. Second, this study surveyed workers at construction sites located in South Korea. Work environment, mental health status, safety awareness, and behavior can be different depending on external factors that vary at the local or national level. Therefore, further research should be performed in other countries and regions with different organizational cultures and work environments.

## 6. Conclusions

This study examined how the construction workers’ perception of the working environment affects their perceptions of motivation, knowledge, and behaviors on work safety through their psychological condition. The hypothetical model was developed based on 11 factors derived from a literature review. Then, by evaluating the fitness indices between the data obtained from 399 construction workers in South Korea and the structural equation model developed based on the hypotheses, the modification model with the best fit was derived. Finally, this study analyzed the direct and indirect effects between the variables and discussed the relationship between the factors using the final model.

The analysis results showed that safety knowledge contributes more than the safety motivation for improving construction works’ safety behavior. Furthermore, construction workers with high levels of depression and anxiety were found to have a low perception of safety behavior. Regarding work environment factors, job demands, lack of job control, lack of reward, and lack of organizational justice had a significantly negative impact on safety behavior, while job support did not influence it.

Through this study, it became clear that various factors related to safety behavior affect it in different ways, and this knowledge will provide better information for construction managers to make an effort to improve the safety outcomes of their sites and the safety behavior of individual workers. However, the number and type of factors discussed in this study are limited. Therefore, it is necessary to analyze them with more diverse factors, such as factors regarding the safety climate and construction sites’ physical environments.

## Figures and Tables

**Table 1:** Questionnaire items on work environment factors.

Variables	Items
X1. Job Demand	X1_01. I work under time pressure. X1_02. I have too much work to do. X1_03. I can have sufficient rest during working hours. (removed) * X1_04. I have to do different works at the same time.
X2. Lack of Job Control	X2_01. I need creativity to do my work * X2_02. I need a high level of skill and knowledge to perform my work * X2_03. I can make a decision with regard to my job * X2_04. I can control the schedule and amount of my work *
X3. Lack of Job Support	X3_01. I can get my supervisor’s help when I need it * X3_02. I can rely upon my co-workers when I feel difficulty doing my work * X3_03. I have someone who understands the difficulties of my work *
X4. Lack of Reward	X4_01. I am worried about my future because my current job is unstable. X4_02. I am afraid undesirable changes are going to happen in my job in the near future. X4_03. I am respected by my company and my co-workers * X4_04. I believe that I will be given more rewards *
X5. Lack of Organizational Justice	X5_01. The organizational policy of my company is fair and reasonable * X5_02. My company provides me with sufficient organizational supports * X5_03. Departments cooperate with each other without conflicts * X5_04. I have opportunities and channels to talk about my ideas. (removed) *

Note: *—reversed score.

**Table 2:** Questionnaire items on depression and trait anxiety.

Variables	Items
M11. Depression	M11_01. I felt depressed. M11_02. I felt that everything I did was an effort * M11_03. I felt hopeful about the future * M11_04. I felt lonely.
M12. Trait Anxiety	M12_01. I feel calm. M12_02. I feel tense. M12_03. I feel upset. M12_04. I feel relaxed. M12_05. I feel content. M12_06. I feel worried.

Note: *—reversed score.

**Table 3:** Questionnaire items on safety motivation, knowledge, and behavior.

Variables	Items
M21.Safety Knowledge	M21_01. I know how to perform my job in a safe manner. M21_02. I know how to use safety equipment and standard work procedures. M21_03. I know how to maintain or improve workplace health and safety. M21_04. I know how to reduce the risk of accidents and incidents in the workplace.
M22. Safety Motivation	M22_01. I believe that workplace health and safety is an important issue. M22_02. I feel that it is worthwhile to put in the effort to maintain or improve my safety. M22_03. I feel that it is important to maintain safety at all times. M22_04. I believe that it is important to reduce the risk of accidents and incidents in the workplace.
Y1. Safety Compliance	Y1_01. I carry out my work in a safe manner. Y1_02. I use all the necessary safety equipment to do my job. Y1_03. I use the correct safety procedures for carrying out my job. Y1_04. I ensure the highest levels of safety when I carry out my job.
Y2. Safety Participation	Y2_01. I promote the safety program within the organization. Y2_02. I put in extra effort to improve the safety of the workplace. Y2_03. I help my co-workers when they are working under risky or hazardous conditions. Y2_04. I voluntarily carry out tasks or activities that help to improve workplace safety.

**Table 4:** Characteristics of samples in completed responses.

Characteristics	Items	N	%
Gender			
	Male	391	98
	Female	8	2.0
Age (years)			
	20–29	12	3.0
	30–39	66	16.5
	40–49	127	31.8
	50~	194	48.6
Contract Type			
	Permanent	49	12.3
	Contract	94	23.6
	Daily	253	63.4
	Other	3	0.01
Career Length (years)			
	0–2 years	41	10.3
	2–5 years	33	8.3
	5–10 years	71	17.8
	10–20 years	123	30.8
	20~	131	32.8
Accident Experience			
	Yes	28	7.0
	No	371	93.0

**Table 5:** Statistics of the research variables.

Construct	Items	Rating	N	Min.	Max.	Mean	SD *
X1. Job demand	3	1–4	399	1.00	4.00	2.48	0.59
X2. Lack of Job control	4	1–4	399	1.00	4.00	2.47	0.47
X3. Lack of Job support	3	1–4	399	1.00	4.00	2.14	0.44
X4. Lack of reward	2	1–4	399	1.00	4.00	2.44	0.61
X5. Lack of Organizational Justice	3	1–4	399	1.00	3.67	2.36	0.48
X6. Job reward	3	1–4	399	1.00	4.00	2.37	0.48
M11. Depression	4	0–3	399	0.00	2.63	0.52	0.50
M12. Trait Anxiety	6	1–5	399	1.00	4.06	2.46	0.54
M21. Safety motivation	4	1–5	399	1.00	5.00	4.07	0.67
M22. Safety knowledge	4	1–5	399	1.00	5.00	3.65	0.71
Y1. Safety compliance	4	1–5	399	1.00	5.00	3.65	0.71
Y2. Safety participation	4	1–5	399	1.75	5.00	3.63	0.66

Note: *—SD: Standard Deviation

**Table 6:** Acceptable threshold levels of fit indices of the structural equation model.

Index Type	Fit Index	Acceptable Threshold Levels
Absolute Fit Indices	Relative χ^2^	<2
	RMSEA	<0.05
	GFI	>0.80
Incremental Fit Indices	NFI	>0.80
	CFI	>0.90

**Table 7:** Results of the validity test of the measurement model.

Constructs	Measures	Estimate	Standard Error	Critical Ratio	P Label	Factor Loading	AVE	Construct Reliability
X1 Lack of Job Demand	X1_01	1.591	0.17	9.379	***	0.877	0.949	0.982
	X1_02	1.28	0.133	9.602	***	0.732		
	X1_03	1				0.532		
X2 Lack of Job Control	X2_01	1.006	0.1	10.031	***	0.670	0.950	0.987
	X2_02	1.127	0.108	10.481	***	0.729		
	X2_03	0.986	0.101	9.806	***	0.647		
	X2_04	1				0.640		
X3 Lack of Job Support	X3_01	1.29	0.101	12.756	***	0.875	0.977	0.992
	X3_02	1.006	0.08	12.543	***	0.771		
	X3_03	1				0.660		
X4 Lack of Reward	X4_01	1.443	0.172	8.415	***	0.683	0.933	0.982
	X4_02	1				0.535		
	X4_03	1.065	0.131	8.114	***	0.628		
	X4_04	1.036	0.135	7.69	***	0.568		
X5 Lack of Organization Justice	X5_01	0.88	0.119	7.389	***	0.506	0.940	0.979
	X5_02	1.164	0.135	8.637	***	0.687		
	X5_03	1				0.620		
M11 Depression	M11_01.	1				0.816	0.957	0.989
	M11_02.	0.883	0.064	13.799	***	0.716		
	M11_03.	0.781	0.065	12.059	***	0.629		
	M11_04.	0.854	0.063	13.503	***	0.700		
M12 Trait Anxiety	M12_01.	0.873	0.074	11.737	***	0.628	0.933	0.988
	M12_02.	0.978	0.076	12.816	***	0.686		
	M12_03.	1				0.725		
	M12_04.	1.03	0.077	13.422	***	0.720		
	M12_05.	1.17	0.079	14.76	***	0.795		
	M12_06.	1.026	0.076	13.574	***	0.728		
M21 Safety motivation	M21_01.	1				0.774	0.977	0.994
	M21_02.	1.012	0.055	18.435	***	0.861		
	M21_03.	0.906	0.051	17.617	***	0.829		
	M21_04.	0.995	0.054	18.581	***	0.867		
M22 Safety knowledge	M22_01.	0.96	0.048	19.969	***	0.807	0.975	0.994
	M22_02.	1.074	0.045	23.741	***	0.893		
	M22_03.	1.091	0.047	23.344	***	0.885		
	M22_04.	1				0.855		
Y1 Safety compliance	Y1_01	1				0.807	0.977	0.994
	Y1_02	1.238	0.063	19.672	***	0.851		
	Y1_03	1.318	0.064	20.58	***	0.879		
	Y1_04	1.202	0.062	19.352	***	0.841		
Y2 Safety participation	Y2_01	1				0.763	0.977	0.994
	Y2_02	0.962	0.06	16.155	***	0.789		
	Y2_03	0.835	0.053	15.729	***	0.771		
	Y2_04	1.047	0.06	17.583	***	0.852		0.982

Note: SD: Standard Deviation; ***—*p* < 0.001

**Table 8:** Correlation matrix of the research variables.

Code	Items	X1	X2	X3	X4	X5	M11	M12	LM21	LM22	LY1	LY2
X1	Lack of Job Demand	1										
X2	Lack of Job Control	−0.136 *	1									
X3	Lack of Job Support	0.17 **	0.331 ***	1								
X4	Lack of Reward	0.354 ***	0.162 *	0.34 ***	1							
X5	Lack of Organization Justice	0.345 ***	0.184 *	0.406 ***	0.607 ***	1						
M11	Depression	0.248 ***	0.008	0.201 **	0.212 **	0.197 **	1					
M12	Trait Anxiety	0.171 **	−0.012	0.2 **	0.369 ***	0.154 *	0.578 ***	1				
M21	Safety motivation	−0.07	−0.093	−0.102 **	−0.089	−0.205 **	−0.315 ***	−0.188 **	1			
M22	Safety knowledge	−0.025	−0.23 ***	−0.12 *	−0.142 *	−0.221**	−0.184 **	−0.184 **	0.699 ***	1		
Y1	Safety compliance	−0.075	−0.183 **	−0.115 *	−0.217 **	−0.323 ***	−0.19 **	−0.182 **	0.568 ***	0.68 ***	1	
Y2	Safety participation	−0.12 *	−0.205 **	−0.164 **	−0.218 **	−0.354 ***	−0.227 ***	−0.185 **	0.583 ***	0.691 ***	0.839 ***	1

Note: *—*p* < 0.05, **—*p* < 0.01, ***—*p* < 0.001

**Table 9:** Standardized direct, indirect, and total effect in the final model.

Endogenous Variables	Exogenous Variables	Total Effect	Direct Effect	Indirect Effect
Y1. Safety compliance	X1. Lack of Job Demand	−0.008		−0.008
	X2. Lack of Job Control	−0.094		−0.094
	X4. Lack of Reward	−0.033		−0.033
	X5. Lack of Organization Justice	−0.190	−0.181	−0.009
	M11. Depression	−0.044		−0.044
	M12. Trait Anxiety	−0.090		−0.090
Y2. Safety participation	X1. Lack of Job Demand	−0.009		−0.009
	X2. Lack of Job Control	−0.093		−0.093
	X4. Lack of Reward	−0.032		−0.032
	X5. Lack of Organization Justice	−0.228	−0.218	−0.010
	M11. Depression	−0.048		−0.048
	M12. Trait Anxiety	−0.089		−0.089
